# RGS16 promotes glioma progression and serves as a prognostic factor

**DOI:** 10.1111/cns.13382

**Published:** 2020-04-22

**Authors:** Ruoyu Huang, Guanzhang Li, Zheng Zhao, Fan Zeng, Kenan Zhang, Yanwei Liu, Kuanyu Wang, Huimin Hu

**Affiliations:** ^1^ Department of Molecular Neuropathology Beijing Neurosurgical Institute Capital Medical University Beijing China; ^2^ Department of Neurosurgery Beijing Tiantan Hospital Capital Medical University Beijing China; ^3^ Chinese Glioma Cooperative Group (CGCG) Beijing China; ^4^ Department of Radiotherapy Beijing Tiantan Hospital Capital Medical University Beijing China; ^5^ Department of Gamma Knife Center Beijing Neurosurgical Institute Capital Medical University Beijing China

**Keywords:** EMT, glioma, immune response, prognosis, RGS16

## Abstract

**Background:**

RGS protein family members have recently became new potentially promising therapeutic targets in many cancers. However, as a key member of RGS family, RGS16 has seldom been studied in glioma. The present study was designed to investigate the prognostic value and biological function of RGS16 based on large‐scale databases and functional assays in vitro.

**Methods:**

Here, we performed comprehensive analysis for the expression characteristic of RGS16 in Chinese Glioma Genome Atlas (CGGA) microarray database with 301 patients and validated in The Cancer Genome Atlas (TCGA) microarray and RNA sequencing database. Student's t‐test, one‐way ANOVA test and long‐rank test were used to assess differences between groups. Kaplan‐Meier survival, univariate and multivariate Cox analysis and ROC curve were used to estimate the survival distributions. Biological implication of abnormal expression of RGS16 in glioma was also explored. Functional analysis of RGS16 was performed in several glioblastoma (GBM) cell lines. R language and SPSS were used for statistical analysis and graphical work.

**Results:**

We found that the expression of RGS16 was positively related to the grade of glioma. High level of RGS16 commonly gathered in glioma of mesenchymal subtype and wild‐type IDH1. Moreover, higher expression level of RGS16 was found to be significantly correlated with poor prognosis. The univariate and multivariate Cox regression analysis and ROC curve showed that RGS16 was an independent prognostic factor for glioma patients. Gene ontology analysis, gene set enrichment analysis, and gene set variation analysis suggested that the overexpression of RGS16 tightly related to cell proliferation, migration, epithelial‐mesenchymal transition (EMT), immune and inflammatory response of glioma. Knockdown of RGS16 in glioma cell lines also showed that RGS16 promoted the malignant progress of glioma cell lines.

**Conclusions:**

RGS16 plays an important role in glioma progression and serves as an independent prognostic factor, especially in GBM patients.

## 1 INTRODUCTION

Glioma is the most common and lethal type of primary brain tumor.^1^, ^2^ Despite the progress of neurosurgery, the prognosis of patients with glioma is still unsatisfactory.^3^, ^4^ Due to the characteristic of infiltrative growth, the tumor tissues is difficult to removed clearly which eventually lead to postoperative recurrence and resistance to both radiotherapy and chemotherapy.^3^ Thus, it is vital to have an insight into the complex regulating mechanism of the progression of this disease, and figure out the critical driver factors which also could be adopted as indicator of survival and worth further investigation as potential therapeutic targets.

The regulators of G protein singling (RGS) gene family members are originally identified as signal transduction G protein‐coupled receptor (GPCRs) inhibitors, due to their ability to increase the intrinsic GTPase activity of G protein.^5^, ^6^ This protein family plays a critical role in the regulation of G protein‐mediated pathways and many other biological processes in various tissues.^7^, ^8^, ^9^ To our knowledge, many RGS family members, including RGS16, act as oncogenes and promote malignancy progression of many human cancers.^10^, ^11^, ^12^, ^13^ Besides, studies focus on RGS protein family also found that RGS16 played central roles in immune and inflammatory responses.^14^, ^15^ However, only a few studies have reported that RGS16 was dysregulated in glioma tissues or cell lines. What's more, the expression pattern and prognostic value of RGS16 in glioma are still unclear.

In this study, we identified RGS16 as a novel prognostic factor in glioma. The Chinese Glioma Genome Atlas (CGGA) microarray dataset was used to evaluate the expression preference, prognostic value, and biological functions of RGS16. Interestingly, we found that RGS16 not only played an important role in malignant progression of glioma, but was also tightly related to the immune and inflammatory response in glioma. These results suggested that RGS16 might serve as a novel immune biomarker of glioma. The above observations were validated in the Cancer Genome Atlas (TCGA) RNA sequencing dataset. Besides, we further detected the roles of RGS16 in several glioma cell lines. All these results indicated that RGS16 was a novel independent prognostic factor and played a crucial role in the malignant progress of glioma. Besides, RGS16 may also serve as a potential therapeutic target in glioma.

## 2 MATERIALS AND METHODS

### 2.1 Patients and samples

The transcriptome data and clinical information of 301 samples from CGGA microarray database (http://www.cgga.org.cn) was used as discovery set. This database was generated by Agilent Whole Human Genome Array platform. TCGA RNA sequencing database and microarray (http://cancergenome.nih.gov) were used as validation set. Before using TCGA database for further analysis, RNA sequencing data were log2‐transformed. In these databases, only samples with definite WHO classification were used for expression analysis and the samples without survival data were excluded in survival analysis.

### 2.2 Cell transfection and Western blot

Human astrocytes (HA) cell line was purchased from ScienCell Research Laboratories. Human glioma cell lines such as H4, U87, LN229, U118 and U251 were purchased from the Institute of Biochemistry and Cell Biology, Chinese Academy of Sciences. DMEM (Gibco; Thermo Fisher Scientific) supplemented with 10% fetal bovine serum (FBS, Gibco; Thermo Fisher Scientific) was used to culture glioma cell lines. HA cell line was cultured in Astrocyte Medium (AM, ScienCell). All the cell lines were maintained in an incubator (37°C, 5% CO2). The RGS16 small interference RNA (siRNA) and negative control (NC) were synthesized by Santa‐cruz Biotechnology Co, Inc. Once cell density reached 30‑50%, LN229, U87 and U251 cell lines were transfected with siRNA or NC (50 nM) at 37˚C using the INTERFERin Transfection reagent (Polyplus‐transfection Co., Ltd.). After 48 hours at 37˚C, fresh medium without siRNA was added to the cells. Western blot analysis was performed with rabbit anti‐RGS16 polyclonal antibody (Abcam, 1:800). Goat anti‐rabbit IgG‐HRP (Abcam, 1:5000) was used as secondary antibodies and β‐tubulin was used for loading control.

### 2.3 Cell scratch assays

U87 and LN229 cells were seeded in 6‐well plates (1 × 10^5 ^cells per well) and incubated in a 37°C, 5% CO2 incubator. At 48 hours after transfected with RGS16 siRNA or a NC siRNA, the cell monolayer was scraped with a sterile 200‐μL pipette tip. Then, fresh medium without serum was added to the plates and each well was photographed to mark the “zero point” of migration. After completion of 48 hours incubation, the samples were washed twice very gently with PBS (Gibco; Thermo Fisher Scientific). Each well was photographed using computer‐assisted microscopy. Phase‐contrast images were taken at the beginning (0 hours) and after 48 hours of incubation for the same scratch area.

### 2.4 Clonogenic assays

LN229 and U251 cells were digested with 0.25% trypsin‐EDTA (Gibco; Thermo Fisher Scientific) when they were in the log phase of growth. Cells were then dissociated and seeded into a 6‐well culture plate. After adherence, cells were transfected with RGS16 siRNA or NC siRNA. Medium was replaced after 8 hours. Plates were maintained in a 37°C, 5% CO2 incubator for 2 weeks. To maintain a low‐level of RGS16 in the whole assay, transfection was repeated at the 7th day after the first transfection. Cell colonies were stained with crystal violet and photographed at the end of after the 2 weeks of incubation.

### 2.5 Cell migration assays

The cell migration assay was performed using 24‐well transwell chambers (Corning). After the transfection with RGS16 siRNA and NC siRNA, 1 × 10^5^ Cells were seeded into upper chambers with 100 μL serum‐free medium and medium with 10% FBS was added into the lower chamber. The LN229 and U251 cells at the bottom surface of filters were fixed and stained with 0.5% crystal violet after 2 hours and 6 hours, respectively.

### 2.6 Bioinformatics analysis

The Pearson correlation analysis was performed to detect genes most closely related to RGS16 expression in CGGA and TCGA datasets. Then, these genes were uploaded to DAVID website (http://david.abcc.ncifcrf.gov/ home.jsp); gene ontology (GO) analysis and Kyoto Encyclopedia of Genes and Genomes (KEGG) pathway analysis were performed to detect the biological processes that correlated with RGS16 expression. Gene set enrichment analysis (GSEA) and gene set variation analysis (GSVA) were used to estimate various biological processes enrichment in each sample.^16^ The immune checkpoints and inflammatory signatures have been described in our previous studies.^17^

### 2.7 Statistical analysis

Kolmogorov‐Smirnov (K‐S) test of normality was used to assess data distribution. Student's *t*‐test and one‐way ANOVA test were used to assess the significance of differences between groups. The prognostic value of RGS16 was estimated by Kaplan‐Meier survival analysis. Univariate and multivariate Cox regression analysis including age, gender, WHO grade, isocitrate dehydrogenase1 (IDH1) mutation status, O‐6‐methylguanine‐DNA methyltransferase (MGMT) methylation status, chemotherapy, and radiotherapy were performed to test independent prognostic factors in SPSS 16.0. Several R packages including ggplot2, survival, pheatmap, pROC, circlize, and corrgram were used for pictures drawing and statistical analysis in R software 3.4.3. In all statistical analysis, a two‐sided *P* value < .05 was considered statistically significant.

## 3 RESULT

### 3.1 RGS16 expression is associated with glioma grade and subtype

**Figure 1:**
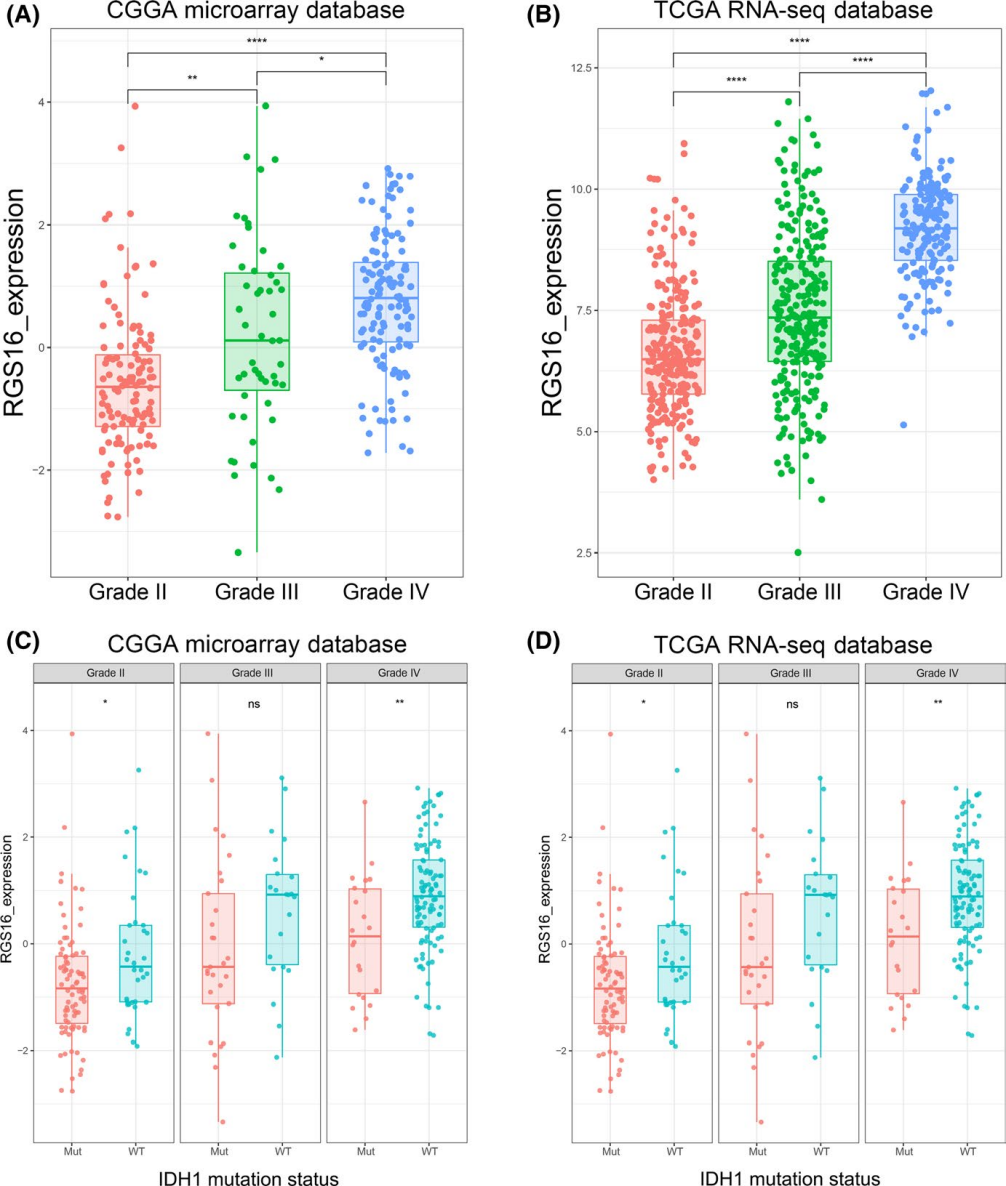
Expression pattern of RGS16 in different grades of gliomas. (A, B) In CGGA microarray and TCGA sequencing database, the mRNA expression level of RGS16 increased with tumor grade. (C, D) In CGGA microarray and TCGA sequencing database, the mRNA expression level of RGS16 was higher in IDH1 wild‐type gliomas than gliomas with mutated IDH1 in each grade, though some groups have no statistically significant. K‐S test of normality was used to assess the distribution of RGS16 expression in CGGA microarray and TCGA sequencing database (*P* = .479 and *P* = .085, respectively). * *P* < .05, ** *P* < .01, **** *P* < .0001, ns: no statistically significant

In consideration of heterogeneity across different grades of glioma, we compared expression levels of differentially expressed genes from the CGGA microarray dataset and found that RGS16 expression was positively correlated with tumor grade. These results were validated in TCGA RNA sequencing and microarray database (Figure 1A,B). After dividing patients into two subgroups according to the IDH1 mutation status, we found that IDH1 mutant‐type showed lower expression of RGS16 across different grades, though some groups have no statistically significant (Figure 1C,D and Figure S1A).

**Figure 2:**
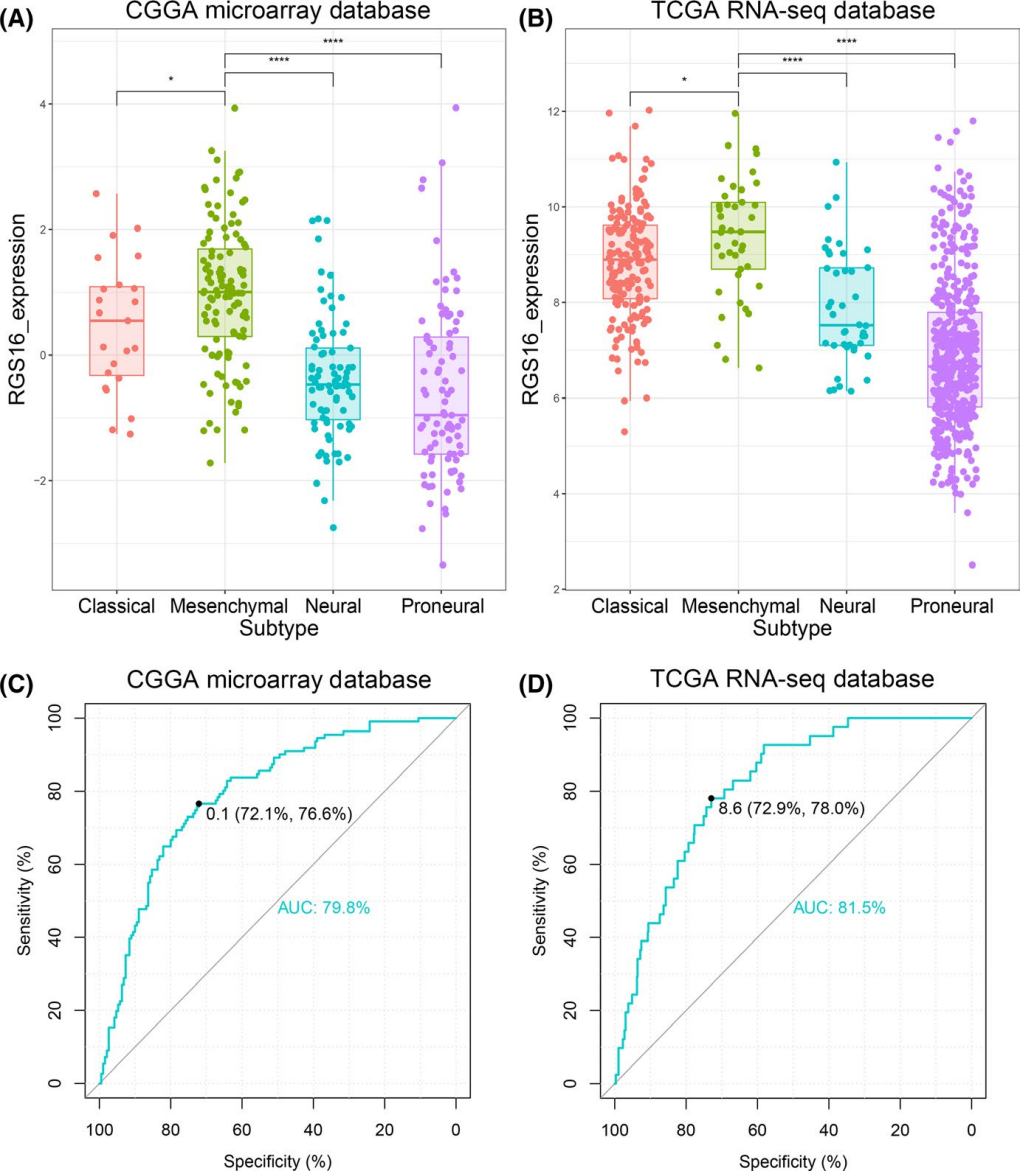
Expression preference of RGS16 in different subtypes of gliomas. (A, B) In CGGA microarray and TCGA sequencing database, RGS16 expression was highest in mesenchymal subtype glioma samples. (C, D) In CGGA microarray and TCGA sequencing database, ROC curve analysis showed that RGS16 has a larger area under the curve in mesenchymal gliomas (AUC is 79.8% and 81.5%, respectively). K‐S test of normality was used to assess the distribution of RGS16 expression in CGGA microarray and TCGA sequencing database (*P* = .479 and *P* = .085, respectively). * *P* < .05, **** *P* < .0001

We analyzed RGS16 expression level among different molecular subtypes defined by TCGA network;^18^ the results showed that the mesenchymal subtype had the highest RGS16 expression in both CGGA and TCGA databases (Figure 2A,B and Figure S1B). These results also suggested that RGS16 had the potential to serve as a biomarker for mesenchymal subtype. To prove it, we performed ROC curves analysis for RGS16 expression and mesenchymal subtype, and the area under curve (AUC) was 79.8% and 81.5% in CGGA microarray and TCGA RNA sequencing database, respectively (Figure 2C,D).

### 3.2 High level of RGS16 predicts poor prognosis in glioma patients

**Figure 3:**
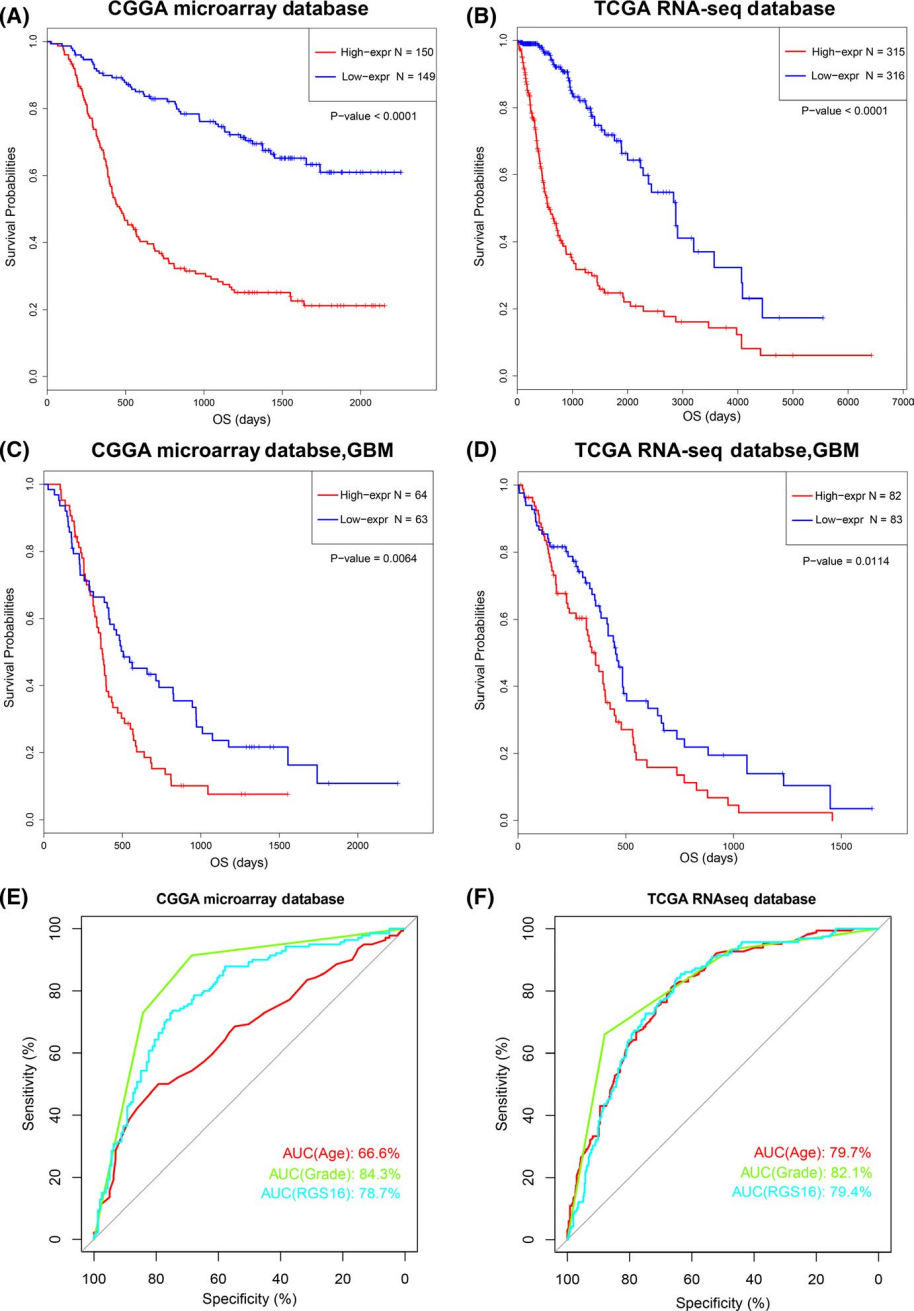
The expression level of RGS16 was correlated to the prognosis of glioma patients. (A, B) In CGGA microarray and TCGA sequencing database, glioma patients with higher expression of RGS16 had a shorter overall survival time. (C, D) In CGGA microarray and TCGA sequencing database, GBM patients with higher expression of RGS16 had a shorter overall survival time. (E, F) The ROC curve showed that the expression level of RGS16 had a large area under the curve in predicting the 3‐year survival rate of glioma patients (AUC is 78.7% and 79.4%, respectively)

To evaluated the association between RGS16 mRNA expression and glioma patients’ clinical outcomes, we performed Kaplan‐Meier (K‐M) survival curve analysis with the data of 299 and 631 patients from the CGGA microarray database and TCGA RNA sequencing database, respectively. The results showed that the overall survival (OS) time of patients with higher RGS16 expression was shorter (Figure 3A,B). Due to the significant biological heterogeneous between GBM and lower grade glioma (LGG), we further investigated the prognostic value of RGS16 in GBM patients in two databases. After dividing patients into two equal groups according to the expression level of RGS16, we found that patients with higher RGS16 expression had a significantly worse prognosis compared with those with lower RGS16 expression (Figure 3C,D). Similar results were also observed in TCGA microarray database (Figure S2). Moreover, in ROC curve analysis, the AUC for RGS16 expression level in predicting the 3 years of survival in CGGA microarray and TCGA RNA sequencing databases were 0.787 and 0.794, respectively (Figure 3E,F). Compared with “age” and “grade,” the AUCs for RGS16 expression level were slightly smaller than those of “grade” in both databases. Those founding mentioned above revealed that RGS16 was a negative prognostic factor in glioma patients.

### 3.3 RGS16 is a novel independent prognostic factor for GBM patients

Considering that the prognosis of patients with GBM was affected by a variety of factors, we performed univariate Cox regression analysis to further estimate the prognostic value of RGS16 in GBM patients of CGGA database. As the results shown in Table 1, RGS16 expression, the age of patients, radiotherapy, and chemotherapy after resection were significantly associated with OS. Thus, a multivariate Cox regression analysis was employed and the result revealed that the expression level of RGS16 was an independent prognostic factor for GBM patients (Table 1). Furthermore, univariate and multivariate Cox regression analysis were also performed with all glioma patients of CGGA database (Table 2). In addition, Cox regression analysis in TCGA RNA sequencing and microarray databases also obtained similar results (Table S1 and S2). These results implied that RGS16 expression might be a novel prognostic biomarker independent of tumor grade and other prognostic factors.

**Table 1:** Univariate and multivariate analysis of OS in CGGA microarray database (GBM)

Variables	Univariate analysis		Multivariate analysis	
	HR (95% CI)	*P* value	HR (95% CI)	*P* value
RGS16 Expression	1.527 (1.202‐1.938)	.001	1.564 (1.190‐2.055)	.001
Age at Diagnosis	1.017 (1.000‐1.034)	.045	1.001 (0.983‐1.020)	.904
Gender	1.205 (0.808‐1.797)	.360		
IDH1 mutation status	0.690 (0.407‐1.172)	.170		
MGMT methylation	0.662 (0.430‐1.020)	.062		
Radiotherapy	0.382 (0.230‐0.634)	<.001	0.433 (0.255‐0.735)	.002
Chemotherapy	0.451 (0.301‐0.675)	<.001	0.549 (0.353‐0.855)	.008

**Table 2:** Univariate and multivariate analysis of OS in CGGA microarray database

Variables	Univariate analysis		Multivariate analysis	
	HR (95% CI)	*P* value	HR (95% CI)	*P* value
RGS16 Expression	1.920 (1.653‐2.230)	<.001	1.406 (1.116‐1.771)	.004
Age at Diagnosis	1.042 (1.027‐1.056)	<.001	1.016 (0.999‐1.034)	.065
Gender	1.222 (0.886‐1.686)	.222	‐	‐
WHO Grade	3.097 (2.507‐3.825)	<.001	2.063 (1.419‐2.998)	<.001
IDH1 mutation status	0.314 (0.221‐0.445)	<.001	0.760 (0.454‐1.273)	.298
MGMT methylation	0.635 (0.438‐0.918)	.016	0.730 (0.492‐1.083)	.118
Radiotherapy	0.585 (0.396‐0.863)	.007	0.429 (0.261‐0.705)	.001
Chemotherapy	1.319 (0.959‐1.816)	.089	‐	‐

### 3.4 RGS16 is associated with the cell proliferation, cell migration and EMT

**Figure 4:**
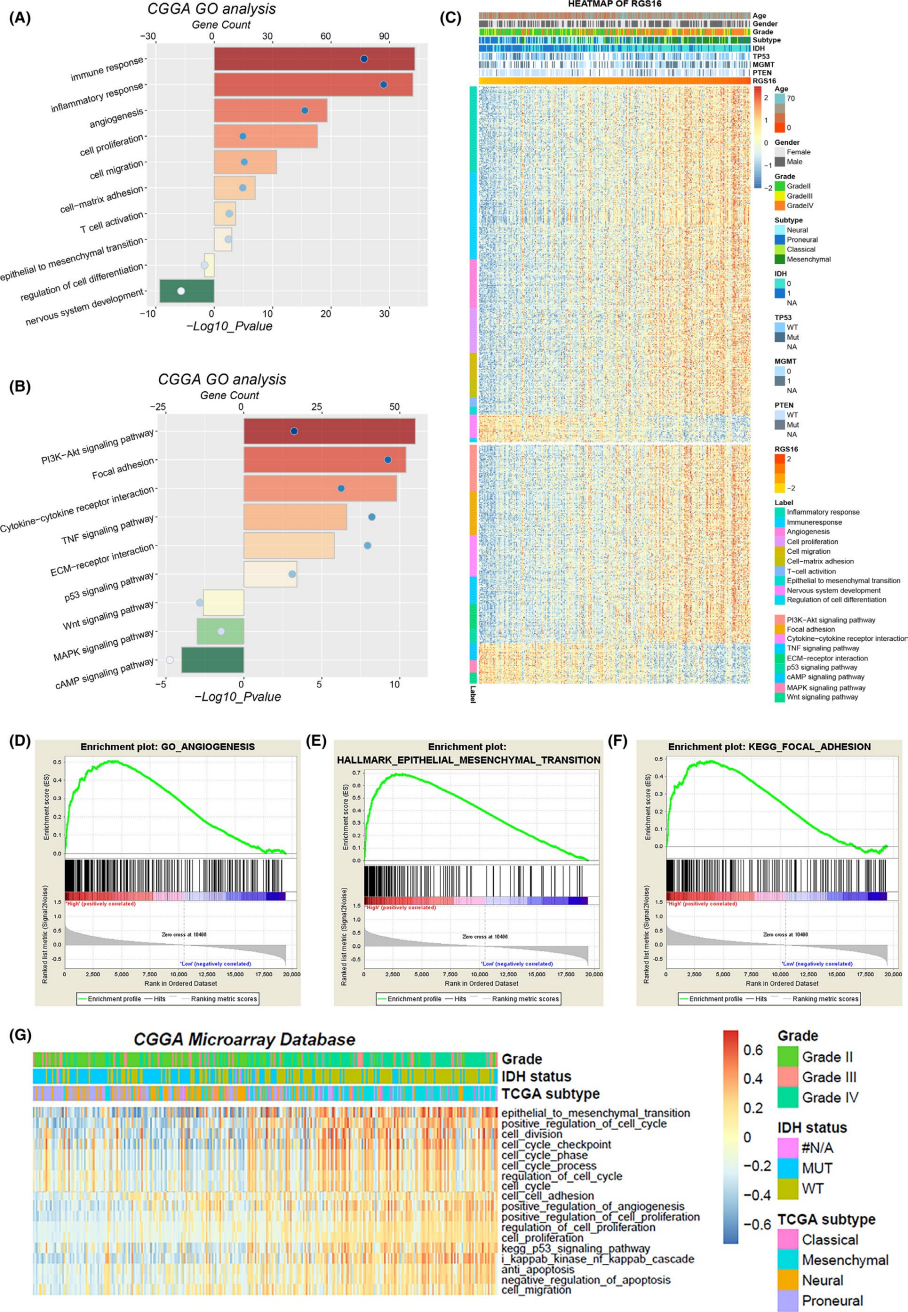
Biological functions related to the expression of RGS16. (A, B, C) In CGGA database, GO analysis and KEGG pathway analysis were performed via the DAVID website to explore the functional annotation of RGS16 related genes. (D, E, F) In CGGA database, the gene set enrichment analysis (GSEA) method was used to explore the tumor‐related biological functions and pathways that were significantly enriched in patients with high level of RGS16 expression. (G) In CGGA database, according to the expression level of RGS16, the degree of enrichment of various biological functions in each sample was analyzed by gene set variation analysis (GSVA)

To further investigate the biological functions of RGS16 in glioma progression, we performed Pearson correlation analysis to find out the genes that tightly correlated with RGS16 expression (Pearson |R| > 0.4) in CGGA and TCGA glioma samples. Then, significantly related genes were used for gene ontology (GO) analysis with DAVID. The results showed that genes that positively correlated with RGS16 expression were enriched in oncogenic processes including immune and inflammatory response, angiogenesis, cell proliferation and migration, T‐cell activation, cell‐matrix adhesion and epithelial to mesenchymal transition（EMT）in GO terms (Figure 4A,C and Figure S3A,C). While genes that negatively correlated with RGS16 trended to enrich in “housekeeping” biological process, such as nervous system development and cell differentiation (Figure 4A,C and Figure S3A,C). The KEGG pathway analysis revealed that RGS16 expression was positively related to PI3K‐AKT signaling pathway and focal adhesion and negatively related to Wnt and cAMP signaling pathway (Figure 4B,C and Figure S3B,C). All the results mentioned above were shared by two databases. Furthermore, gene set enrichment analysis (GSEA) uncovered similar results (Figure 4D,E and F). To get more accurate results, we also performed gene set variation analysis (GSVA) to further evaluate the enrichment score of biological process and pathways in each sample (Figure 4G and Figure S3D). These analyses indicated that RGS16 might play a key role in the malignant progression of glioma.

### 3.5 RGS16 is tightly correlated with T‐cell immunity in glioma

**Figure 5:**
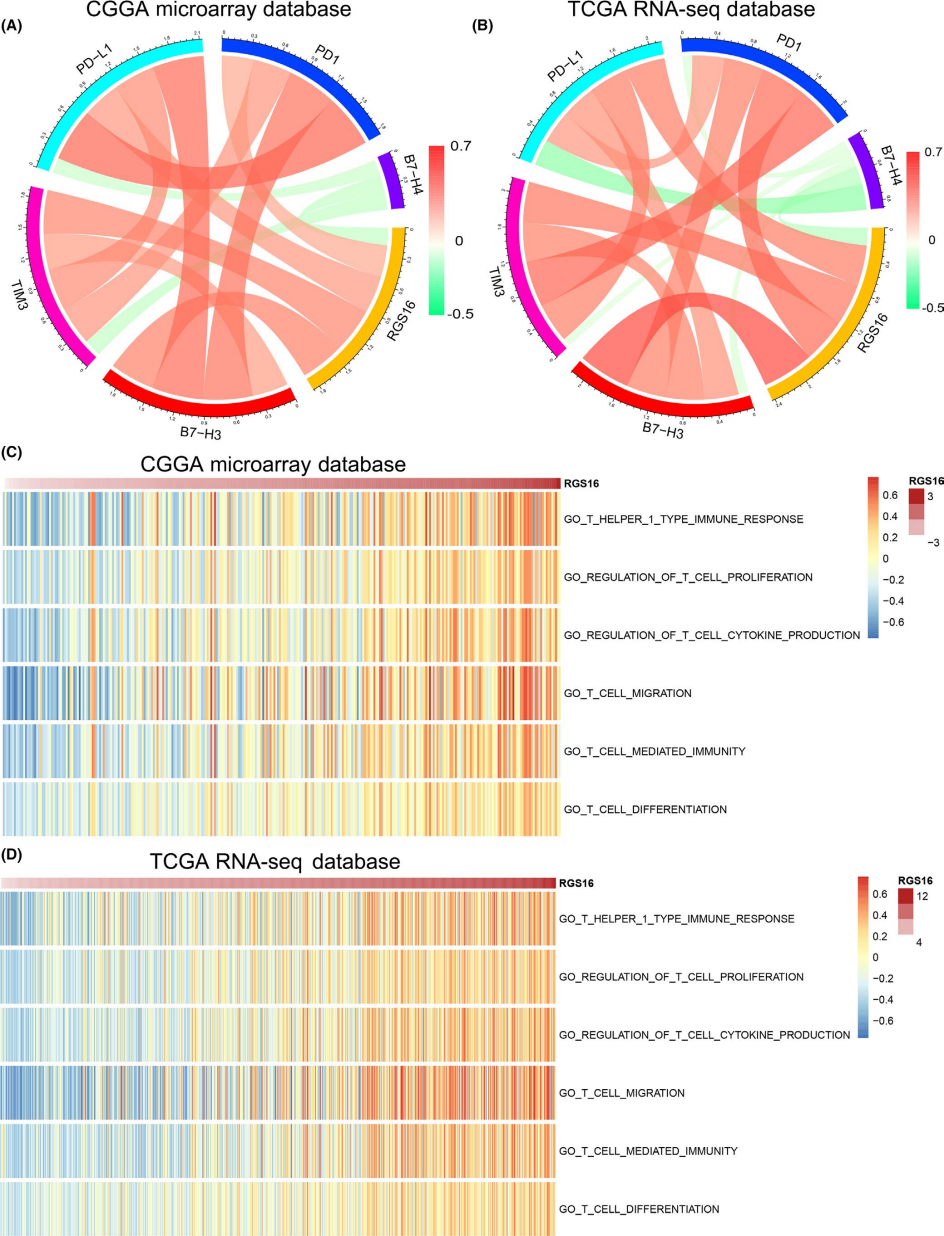
The association between RGS16 expression and immune responses. (A, B) In CGGA and TCGA databases, the relationship between RGS16 expression level and immune checkpoints was explored by CIRCOS analysis. (C, D) In CGGA and TCGA databases, the correlation between the expression level of RGS16 and the T cell–mediated immune response was analyzed by gene set variation analysis (GSVA)

According to the results of the GO analysis, genes that tightly correlated with RGS16 expression were significantly enriched in immune and inflammatory response, as well as T‐cell activation. These results indicated that RGS16 may play an essential role in tumor‐induced immune and inflammatory process. To further investigate the RGS16‐related immune activation, key members of immune checkpoint were chosen to analysis together with RGS16. CIRCOS plots revealed that RGS16 was positively correlated with PD1, PD‐L1, and TIM3 (Figure 5A,B). This result indicated that the overexpression of RGS16 was tightly associated with PD1/PD‐L1 pathway activation. TIM3 was reported to have special immune functions in glioma, especially T cell–related immune response.^19^ The relationship between TIM3 and RGS16 reminded us that RGS16 might also play a key role in T‐cell immune process. To verify this, GSVA analysis was performed. The results showed that most of T lineage–related immune responses were positively correlated with RGS16 expression (Figure 5C,D).

### 3.6 RGS16 is associated with inflammatory activities in gliomas

**Figure 6:**
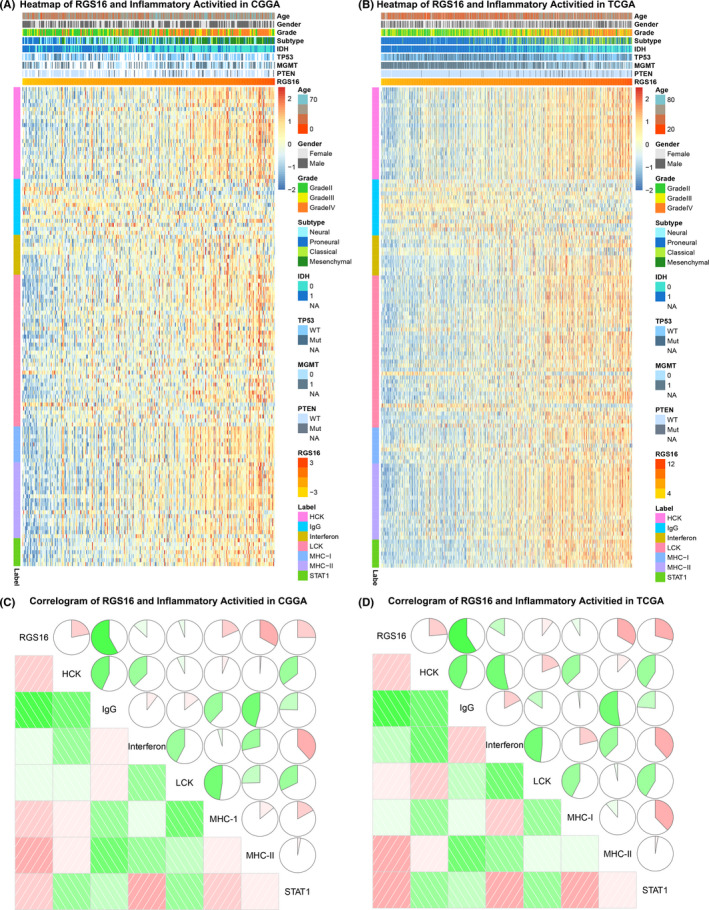
The association between RGS16 expression level and biomarkers of inflammatory response. (A, B) The relationship between classic inflammatory signatures and RGS16 expression levels was explored in CGGA and TCGA databases. (C, D) In two databases, Corrgram analysis was used to explore the relationship between inflammatory signatures and RGS16 expression levels

To explore the role of RGS16 in inflammatory activation in gliomas, we chose seven inflammatory metagenes described in previous studies to represent different inflammation response.^17^ As showed by the results, most of these signatures were positively related to RGS16, while IgG was quite the opposite (Figure 6A,B). Perhaps it meant that RGS16 was tightly correlated with macrophages and T cell–related inflammatory response rather than B lymphocyte. Correlogram analysis was performed to further validate what we found in cluster analysis. The results also indicated that RGS16 was not involved in B lineage‐related inflammatory responses (Figure 6C,D). Similar findings were also reported in previous researches on PD1 and TIM‐3.^17^, ^19^

### 3.7 RGS16 promotes glioma progression in vitro

**Figure 7:**
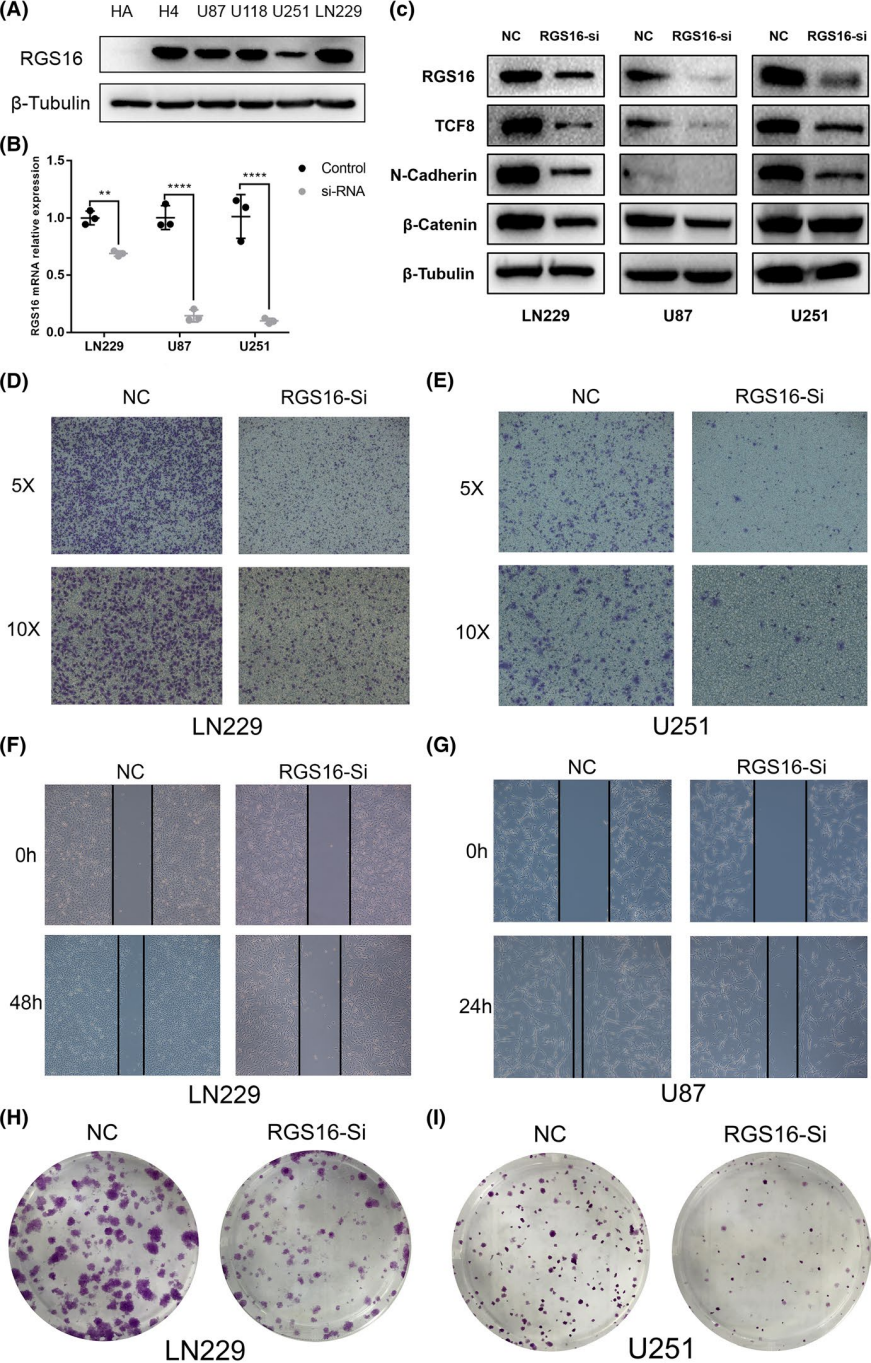
RGS16 played an oncogene role in glioma cell lines. (A) The expression of RGS16 protein in the human astrocytes (HA) cell line and glioma cell lines. (B) The real‐time quantitative PCR (qPCR) assay of RGS16 mRNA expression in LN229, U87, and U251 cell lines after infection with RGS16 siRNA or negative control. (C) The Western blot analysis of RGS16, TCF8, N‐cadherin, and β‐catenin protein expression in LN229, U87, and U251 cell lines after applying RGS16 siRNA or negative control. (D, E) Transwell assay of LN229 and U251 cell lines treated with RGS16 siRNA or negative control. (D) Cell scratch assay of LN229 and U87 cell lines treated with RGS16 siRNA or negative control. (E) Clonogenic assay of LN229 and U251 cell lines treated with RGS16 siRNA or negative control

As the results showed in GO analysis, the high level of RGS16 expression was positively correlated with cell proliferation, cell migration, and epithelial to mesenchymal transition (EMT). To further confirm these functional roles of RGS16 in glioma, we performed experiments in vitro. Firstly, we detected RGS16 protein levels in H4, LN229, U87, U251, U118 glioma cell lines and human astrocytes (HA) cell line; the Western blot analysis indicated that LN229 had the highest expression level while HA had the lowest (Figure 7A). Then, RGS16 small interference RNA (siRNA) was transfected to knockdown RGS16 expression in LN229, U251, and U87 cell lines (Figure 7B,C). After that, we detected the protein levels of several EMT signaling pathway biomarkers; Western blot analysis showed that the expression level of TCF8, N‐cadherin, and β‐catenin protein in three cell lines has dropped in varying degrees (Figure 7C). These results suggested that RGS16 has a significant impact on the EMT signaling pathway. Furthermore, to evaluate the function of RGS16 in glioma cell lines, transwell and cell scratch were performed. The results indicated that the silence of RGS16 expression suppressed the migration ability of glioma cell lines (Figure 7D,E,F, and G), Besides, the clonogenic assay showed that the proliferative capacity and clonogenicity of glioma cell lines were inhibited after transfection with RGS16 siRNA (Figure 7H,I).

## 4 DISCUSSION

Due to the rapidly proliferation and intensive invasion behaviors, gliomas became the deadliest intracranial tumor.^1^ Prognosis of patients with glioma remains poor despite the radiotherapy and chemotherapy combine with surgical resection. Thus, there is a pressing need for new therapeutic approaches. Various tumor‐specific molecular alterations have been thoroughly studied in glioma, such as isocitrate dehydrogenase 1 (IDH1) mutations,^20^ O6‑methylguanine‐DNA methyltransferase (MGMT) promoter methylation,^21^ 1p/19q co‑deletion, and epidermal growth factor receptor variant III (EGFR vIII) amplification, which have been identified as predictive and prognostic indicators and/or therapeutic targets for patients with glioma.^22^, ^23^ Moreover, in recent years, with the constant development and improvement of immunotherapy, more and more patients with malignant tumor benefit from it.^24^, ^25^ However, the side effects of immunotherapy make it difficult to be widely applied. To change this situation, we should further explore the mechanism of immunotherapy. In this study, we believe that RGS16 played an important role in malignant progress of glioma and had potential to become a novel immune‐related biomarker.

According to former research achievements, RGS16 was involved in a variety of cellular functions and signal regulation. For example, RGS16 could negatively regulate platelet function and thrombosis, and chemokine CXCL12 also regulates platelet activation by affecting RGS16 expression.^26^ In addition, RGS16 also participates in the process of glucose and lipid metabolism.^27^, ^28^ Relevant studies have confirmed that RGS16 promotes the proliferation of beta cells and insulin secretion in human and mouse islets.^27^ Besides, RGS16 also plays an important role in immune and inflammatory response. Firstly, related studies have confirmed that RGS16 protein can regulate T lymphocyte‐mediated inflammatory response by affecting the activation of T lymphocytes and promote T lymphocyte migration via chemokines such as CXCR4, CCR3, and CCR5.^29^, ^30^ In addition, RGS16 also can negatively regulate monocyte‐mediated inflammatory response by inhibiting the monocytes‐related inflammatory cytokines.^15^

RGS16 exerts different biological functions in a variety of benign and malignant tumors. In some patients with breast cancer, deletion of RGS16 promotes the activation of PI3K signaling pathway by growth factors and thus promotes tumor proliferation, HER2 activation, and resistance to chemotherapeutic drugs.^31^ Conversely, high expression of RGS16 can inhibit breast cancer growth via the PI3K signaling pathway. Related researches revealed that RGS16 could inhibit the migration and invasion of pancreatic cancer cell lines in vitro by interacting with p53 and pRB.^32^ Meanwhile, previous studies have found that the expression levels of RGS16 and FosB were significantly reduced in pancreatic cancer with lymph node metastasis.^33^ These findings suggested that RGS16 is closely associated with the progression of prostate cancer and may serve as an independent prognostic factor for patients with pancreatic cancer. In colorectal cancer, RGS16 exerts completely different biological functions. The expression level of RGS16 mRNA and protein in colorectal cancer tissues were much higher than that in normal tissues. The prognosis of patients with high expression of RGS16 was significantly worse than patients with low RGS16 expression.^34^

In this study, we detected the molecular and clinical characterization of RGS16 through more than 1000 glioma samples from TCGA and CGGA databases. We found that the expression level of RGS16 was positively correlated with tumor grade and significantly upregulated in mesenchymal subtype, which is generally considered to have a poor prognosis. Moreover, high level of RGS16 also significantly preferred to IDH1 wild‐type samples. K‐M survival curve analysis and ROC curve analysis indicated that RGS16 was a novel prognostic factor in glioma patients. These results were further confirmed by univariate and multivariate Cox regression analysis. All these results revealed that RGS16 expression tightly associated with malignant progression of gliomas, which has been reported in other solid cancers. Besides, as a regulator of G protein signaling pathway, RGS16 also played an important role in the regulation of immunity and inflammatory responses. To study the immune function of RGS16 in glioma can help us better understand and design the immunotherapy of glioma.

Although, in this study, the functions of RGS16 in gliomas were deeply analyzed and discussed, the relationship between RGS16 expression and the biological characteristics of glioma remains unclear. The in vitro experiments conducted in this study still need to be improved. Further understanding of the function role and expression pattern of RGS16 in gliomas may provide more accurate diagnosis and prognosis predictions of patients, as well as the improvement of personalized therapeutics of glioma.

## 5 CONCLUSION

In conclusion, we found that RGS16 plays an important role in the malignant progression of glioma. Bioinformatics analyses showed that RGS16 was involved in cell proliferation, migration, EMT, and immune and inflammatory response of glioma. Experiments in vitro suggested that RGS16 was overexpressed in glioma cell lines and promoted cell proliferation and migration via EMT process. Thus, we believe that RGS16 had the potential to serve as a novel prognostic biomarker and therapy target.

## CONFLICT OF INTEREST

The authors declare that they have no conflict of interests.

## ETHICAL APPROVAL

This study was approved by the institutional review board (IRB) of Beijing Tiantan Hospital, and informed consent was obtained from all individual participants for CGGA project in this study.

## Supporting information

Figure S1

Figure S2

Figure S3

Table S1

Table S2
