# Corrigendum

**DOI:** 10.1111/cns.13844

**Published:** 2022-04-12

In Huang et al^1^ the authors noticed that the same figure was accidently presented in Figure 1C and 1D. In addition, the authors also found the Figure 7 caption to be incorrect.

**FIGURE 1:**
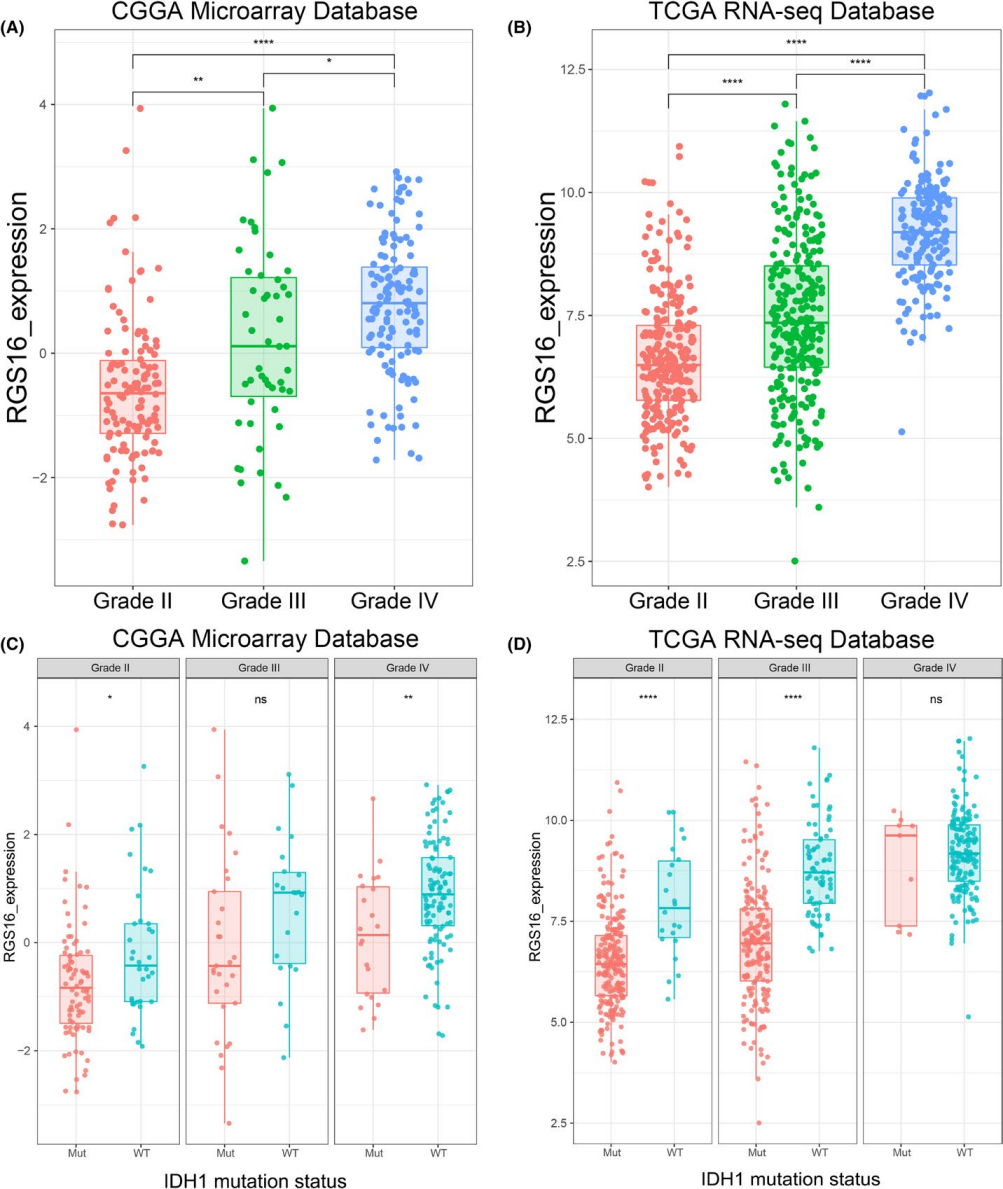
Expression pattern of RGS16 in different grades of gliomas. (A,B) In CGGA microarray and TCGA sequencing database, the mRNA expression level of RGS16 increased with tumor grade. (C,D) In CGGA microarray and TCGA sequencing database, the mRNA expression level of RGS16 was higher in IDH1 wildtype gliomas than gliomas with mutated IDH1 in each grade, though some groups have no statistically significant. K–S test of normality was used to assess the distribution of RGS16 expression in CGGA microarray and TCGA sequencing database (*p* = 0.479 and *p* = 0.085, respectively). * *p* < 0.05, **p < 0.01, **** *p* < 0.0001, ns: no statistically significant

The correct Figure 1 is given below:

The correct Figure 7 legend is given below:

Figure 7. RGS16 played an oncogene role in glioma cell lines. (A) The expression of RGS16 protein in the human astrocytes (HA) cell line and glioma cell lines. (B) The real‐time quantitative PCR (qPCR) assay of RGS16 mRNA expression in LN229, U87 and U251 cell lines after infection with RGS16 siRNA or negative control. (C) The western blot analysis of RGS16, TCF8, N‐cadherin and β‐catenin protein expression in LN229, U87 and U251 cell lines after applying RGS16 siRNA or negative control. (D, E) Transwell assay of LN229 and U251 cell lines treated with RGS16 siRNA or negative control. (F, G) Cell scratch assay of LN229 and U87 cell lines treated with RGS16 siRNA or negative control. (H, I) Clonogenic assay of LN229 and U251 cell lines treated with RGS16 siRNA or negative control.

It earlier read as,

RGS16 played an oncogene role in glioma cell lines. (A) The expression of RGS16 protein in the human astrocytes (HA) cell line and glioma cell lines. (B) The real‐time quantitative PCR (qPCR) assay of RGS16 mRNA expression in LN229, U87, and U251 cell lines after infection with RGS16 siRNA or negative control. (C) The Western blot analysis of RGS16, TCF8, N‐cadherin, and β‐catenin protein expression in LN229, U87, and U251 cell lines after applying RGS16 siRNA or negative control. (D, E) Transwell assay of LN229 and U251 cell lines treated with RGS16 siRNA or negative control. (D) Cell scratch assay of LN229 and U87 cell lines treated with RGS16 siRNA or negative control. (E) Clonogenic assay of LN229 and U251 cell lines treated with RGS16 siRNA or negative control.

The authors apologize for these errors.

## References

[1] Huang R , Li G , Zhao Z , et al. RGS16 promotes glioma progression and serves as a prognostic factor. CNS Neurosci Ther. 2020;26:791‐803. 32319728 10.1111/cns.13382 PMC7366748

