# Supplementary figures and images for: RGS16 promotes glioma progression and serves as a prognostic factor
Source: CNS Neurosci Ther. 2020 Apr 22;26(8):791–803. doi: 10.1111/cns.13382 (PMC7366748; doi:10.1111/cns.13382)

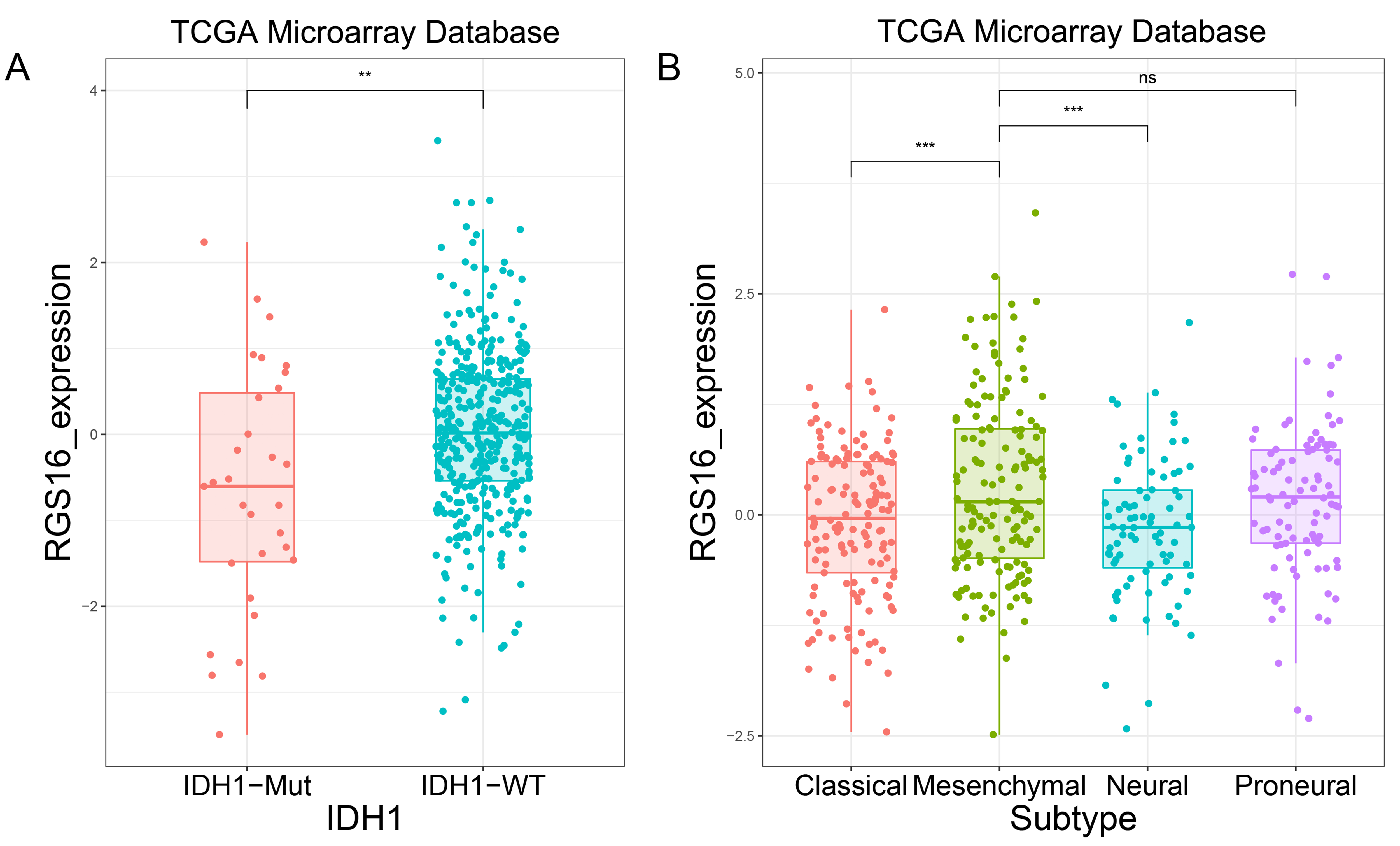

Supplement: Supplementary file 1 — Figure S1 [file CNS-26-791-s001.tif]

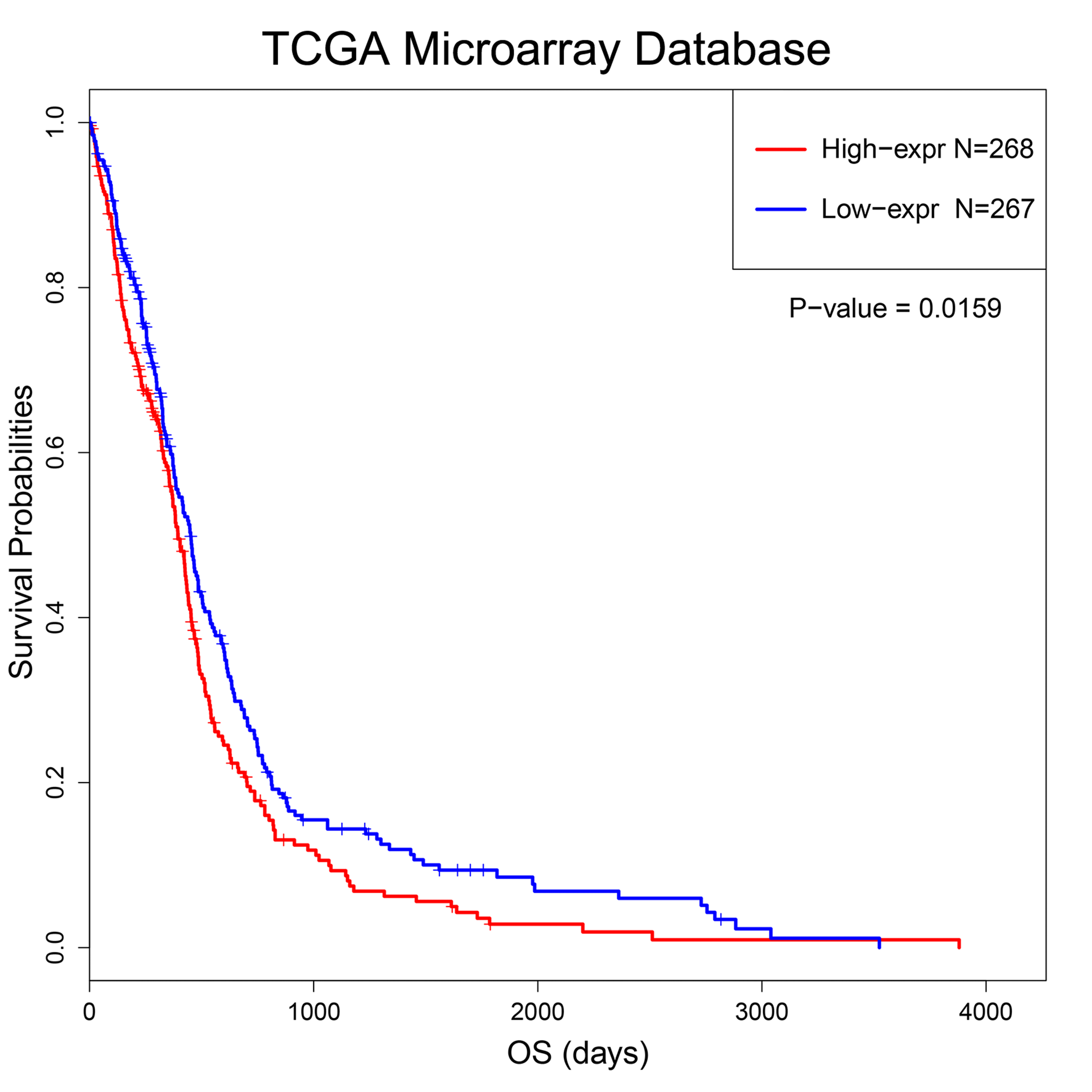

Supplement: Supplementary file 2 — Figure S2 [file CNS-26-791-s002.tif]

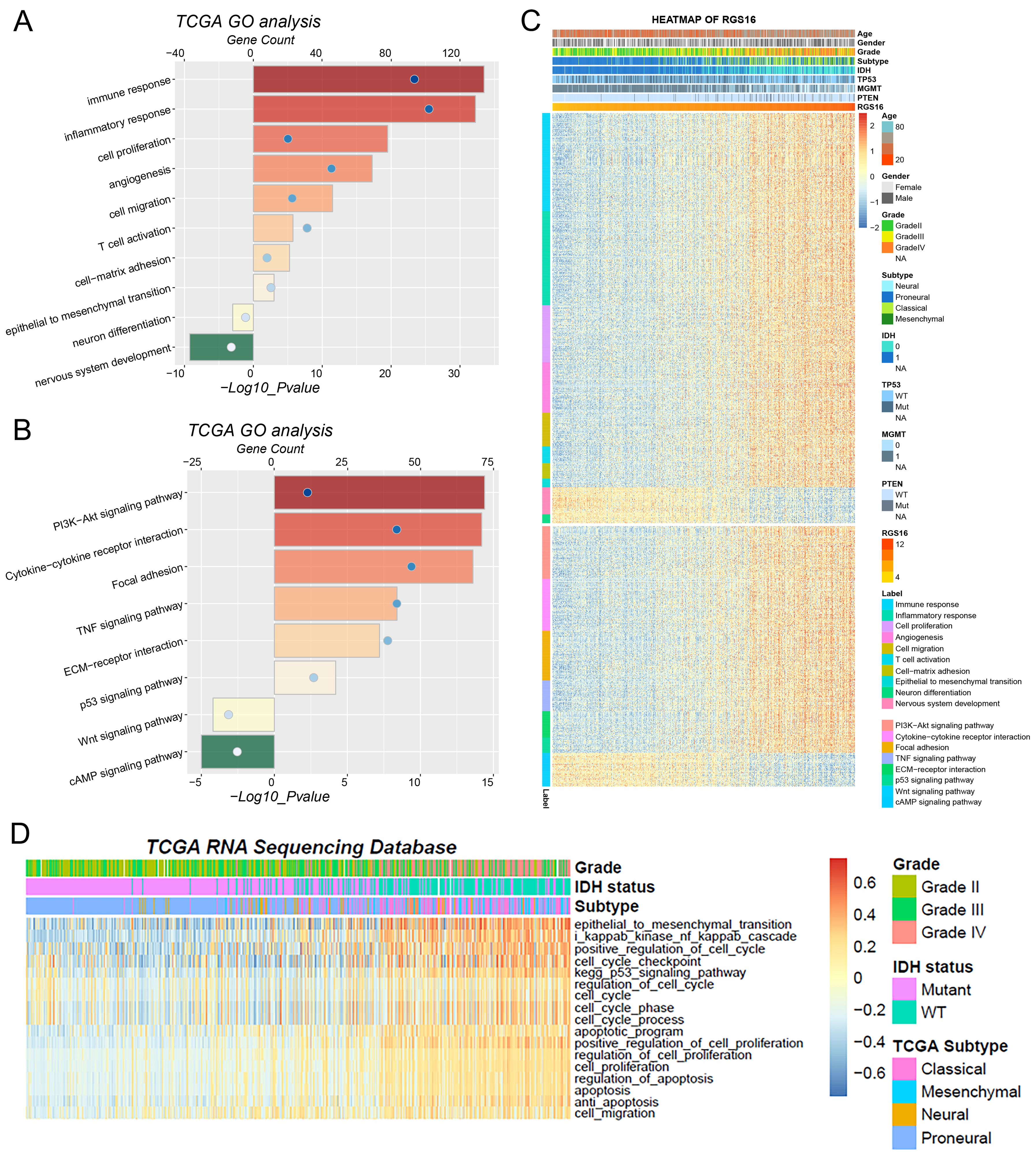

Supplement: Supplementary file 3 — Figure S3 [file CNS-26-791-s003.tif]
