# Supplementary material for: RGS16 promotes glioma progression and serves as a prognostic factor
Source: CNS Neurosci Ther. 2020 Apr 22;26(8):791–803. doi: 10.1111/cns.13382 (PMC7366748; doi:10.1111/cns.13382)
Supplement: Supplementary file 4 — Table S1 [file CNS-26-791-s004.docx]

**Supplementary Table I. Univariate and multivariate analysis of OS in TCGA RNAseq database**

| **Variables** | **Univariate analysis** | | **Multivariate analysis** | |
| --- | --- | --- | --- | --- |
|  | **HR (95% CI)** | **p value** | **HR (95% CI)** | **p value** |
| **RGS16 Expression** | 1.589 (1.463-1.725) | < 0.001 | 1.216 (1.063-1.390) | 0.004 |
| **Age at Diagnosis** | 1.077 (1.065-1.088) | < 0.001 | 1.041 (1.023-1.060) | < 0.001 |
| **Gender** | 1.104 (0.828-1.472) | 0.499 |  |  |
| **WHO Grade** | 4.845 (3.860-6.081) | < 0.001 | 1.925 (1.259-2.943) | 0.002 |
| **IDH1 mutation status** | 0.127 (0.094-0.173) | < 0.001 | 0.679 (0.359-1.284) | 0.234 |
| **KPS** | 0.954 (0.943-0.965) | < 0.001 | 0.988 (0.974-1.002) | 0.099 |
