# Supplementary material for: RGS16 promotes glioma progression and serves as a prognostic factor
Source: CNS Neurosci Ther. 2020 Apr 22;26(8):791–803. doi: 10.1111/cns.13382 (PMC7366748; doi:10.1111/cns.13382)
Supplement: Supplementary file 5 — Table S2 [file CNS-26-791-s005.docx]

**Supplementary Table II. Univariate and multivariate analysis of OS in TCGA microarray database**

| **Variables** | **Univariate analysis** | | **Multivariate analysis** | |
| --- | --- | --- | --- | --- |
|  | **HR (95% CI)** | **p value** | **HR (95% CI)** | **p value** |
| **RGS16 Expression** | 1.152 (1.059-1.254) | 0.001 | 1.197 (1.069-1.340) | 0.002 |
| **Age at Diagnosis** | 1.036 (1.028-1.044) | < 0.001 | 1.023 (1.012-1.033) | < 0.001 |
| **Gender** | 1.047 (0.858-1.278) | 0.652 |  |  |
| **IDH1 mutation status** | 0.351 (0.220-0.559) | < 0.001 | 0.518 (0.309-0.871) | 0.013 |
| **Radiotherapy** | 0.220 (0.120-0.405) | < 0.001 | 0.167 (0.086-0.325) | < 0.001 |
| **Chemotherapy** | 0.353 (0.284-0.438) | < 0.001 | 0.335 (0.255-0.441) | < 0.001 |
